# Predisposing conditions for condylar sag after intraoral vertical ramus osteotomy

**DOI:** 10.1038/s41598-021-89968-w

**Published:** 2021-05-17

**Authors:** Satoshi Rokutanda, Shin-Ichi Yamada, Souichi Yanamoto, Hiroshi Sakamoto, Keisuke Omori, Hiromi Rokutanda, Tomoko Yoshimi, Ayumi Fujishita, Yukiko Morita, Noriaki Yoshida, Masahiro Umeda

**Affiliations:** 1 Department of Clinical Oral Oncology, Nagasaki University Graduate School of Biomedical Sciences, 1-7-1 Sakamoto, Nagasaki City, Nagasaki 852-8588 Japan; 2 Department of Oral and Maxillofacial Surgery, Juko Memorial Nagasaki Hospital, 6-17 Maruo Town, Nagasaki City, Nagasaki 852-8004 Japan; 3 Department of Dentistry and Oral Surgery, Shinshu University School of Medicine, 3-1-1 Asahi, Matsumoto City, Nagano 390-8621 Japan; 4 Department of Orthodontics and Dentofacial Orthopedics, Nagasaki University Graduate School of Biomedical Sciences, 1-7-1 Sakamoto, Nagasaki City, Nagasaki 852-8588 Japan

**Keywords:** Medical research, Risk factors

## Abstract

Intraoral vertical ramus osteotomy (IVRO) is used to treat mandibular prognathism and temporomandibular disorders. However, the improvement of temporomandibular disorders after IVRO is considered to be due to the anterior and downward movement of the mandibular condyle, which may lead to condylar sag, and in the worst case, condylar luxation. In this retrospective cohort study, we examined factors potentially associated with condylar sag. Univariate analysis indicated that condylar sag was significantly associated with the following factors: magnitude of setback (*P* = 0.001), less than 3 mm setback (*P* < 0.001), presence of temporomandibular joint (TMJ) symptoms (*P* = 0.002), Wilkes classification (*P* = 0.039), occlusal cant correction ≥ 2 mm (*P* = 0.018), and mandibular condyle deformation (*P* < 0.001). Setback magnitude (*P* = 0.032) and TMJ symptoms (*P* = 0.007) remained significant in the multivariate analysis. In the receiver operating characteristic curve, the setback magnitude cut-off value for condylar sag after IVRO was 3.25 mm. Thus, the incidence of condylar sag after IVRO is increased with a smaller setback magnitude (≤ 3.25 mm) and the presence of TMJ symptoms. These factors should be evaluated by surgeons during treatment planning for IVRO to estimate condylar sag, and it may be possible to predict the risk of condylar luxation.

## Introduction

Intraoral vertical ramus osteotomy (IVRO) and sagittal split ramus osteotomy (SSRO) have been used extensively to correct mandibular prognathism, and IVRO is one of the treatment modalities for certain types of temporomandibular joint disorders (TMDs)^1–3^. IVRO is effective in alleviating TMD symptoms^4^ and is also advantageous in that it is technically simple^5^, facilitates the repositioning of the condyle^5–7^, and is associated with a lower incidence of inferior alveolar nerve injury^8^. On the other hand, IVRO has some disadvantages compared to the SSRO. First, the principal disadvantage of the IVRO is the need for maxillomandibular fixation (MMF)^5^. Second, depending on the magnitude of setback, posterior drift may occur after IVRO, which adversely affects the corrective treatment^9^. The most pertinent issue for the surgeon is the difficulty in predicting the magnitude of condylar sag that may occur after IVRO. Indeed, severe cases of condylar luxation have been reported to occur unexpectedly after IVRO^10^.

Patients with TMD are more prone to temporomandibular luxation after IVRO compared to patients with jaw deformities^10^. Methods for preventing this complication have been proposed. Kawase-Koga et al.^11^ reported that condylar luxation can be prevented by adjusting the osteotomy line shape. Rotskoff et al.^12^, on the other hand, described the use of an overcorrected occlusal splint to avoid condylar displacement. Nevertheless, the optimal method for managing condylar luxation after it has occurred remains unclear.

Condylar sag improves temporomandibular joint (TMJ) symptoms, which will further decrease over time^4^. The appropriate treatment for condylar luxation is controversial, with some studies advocating the use of conservative management^10^, and other studies reporting the need for surgical treatment^13^. As condylar luxation is thought to occur continuously during condylar sag^2^, we investigated the conditions under which condylar sag was likely to occur.

## Results

The total number of included patients was 57, and 114 TMJs were investigated. No patients were excluded by the exclusion criteria. The mean age of the cohort (21 male and 36 female patients) at the time of surgery was 27.2 ± 10.4 (range: 17 to 54) years. The mean setback achieved with IVRO was 5.9 ± 3.4 mm (range: 0 to 15 mm). Condylar sag was diagnosed in 42 of 114 TMJs (37%), while condylar luxation was only diagnosed in one TMJ (0.87%). Approximately a third (39 of 114) of the TMJs exhibited symptoms. Maxillary surgery was performed in 26 of 57 cases (46%); concurrent occlusal cant correction was carried out in 14 of 26 cases (54%), with the majority involving corrections of 2 mm or more (12 of 26 cases). Mandibular condyle deformation was observed in 12 of 144 (11%) TMJs. Condylar sag was observed in 20 of the 75 (27%) joints that did not exhibit symptoms. Condylar sag was observed in 22 of the 39 (56%) joints that exhibited symptoms. In terms of the Wilkes classification^14^, 33 (29%), 5 (4%), and 2 (2%) of the 114 TMJs were classified as being in the early, early/intermediate, and intermediate/late stages of internal derangement, respectively. Seventy-four of the 114 (65%) TMJs did not exhibit clinical features of internal derangement. The maximal mouth opening was 30 mm or less for 6 (5%) TMJs. Pain (visual analog scale score of 3 or more) on opening or closing the mouth was reported in 8 (7%) TMJs. Habitual luxation was observed in 2 (2%) TMJs. (Table 1).

**Table 1:** Summary of patient data.

**Mean age at the time of surgery**		
27.2 ± 10.4 years		
**Sex**		
Male: 21		
Female: 36		
**Magnitude of setback**		
5.9 ± 3.4 mm		
(0–15 mm)		
**Condylar sag or luxation**		
Sag: 42 of 114 temporomandibular joints (37%)		
Luxation: 1 of 114 temporomandibular joints (0.87%)		
**Temporomandibular joint symptoms**		
39 of 114 temporomandibular joints (34%)		
**Maxillary surgery**	**Maxillary surgery with an occlusal cant correction**	**Maxillary surgery with an occlusal cant correction of 2 mm or more**
26 of 57 cases (46%)	14 of 26 cases (54%)	12 of 26 cases (46%)
**Mandibular condyle deformation**		
12 of 114 temporomandibular joints (11%)		
**No temporomandibular joint symptoms, with condylar sag**		**With temporomandibular joint symptoms, with condylar sag**
20 of 75 temporomandibular joints (27%)		22 of 39 temporomandibular joints (56%)
**Wilkes classification**		**Maximal mouth opening**
No findings: 74 of 114 temporomandibular joints (65%)		more than 30 mm: 108 of 114 temporomandibular joints (95%)
Early: 33 of 114 temporomandibular joints (29%)		30 mm or less: 6 of 114 temporomandibular joints (5%)
Early/intermediate: 5 of 114 temporomandibular joints (4%)		
Intermediate: 0 of 114 temporomandibular joints		**Pain on mouth opening or closing**
Intermediate/late: 2 of 114 temporomandibular joints (2%)		(−): 106 of 114 temporomandibular joints (93%)
Late: 0 of 114 temporomandibular joints		(+): 8 of 114 temporomandibular joints (7%)
		**Habitual luxation of temporomandibular joint**
		(−): 112 of 114 temporomandibular joints (98%)
		(+): 2 of 114 temporomandibular joints (2%)

Univariate analyses of potential explanatory factors for condylar sag after IVRO did not find significant associations for age, sex, classification of the main and sub-functional side of the TMJ, maxillary surgery, occlusal cant modification, osteotomy line shape, habitual TMJ luxation, a maximum mouth opening of 30 mm or less, or pain on opening or closing the mouth. On the other hand, significant associations were found for the magnitude of setback (*P* = 0.001), setback < 3 mm (*P* < 0.001), presence of TMJ symptoms (*P* = 0.002), internal derangement as per Wilkes classification (*P* = 0.039), occlusal cant correction ≥ 2 mm (*P* = 0.018), and mandibular condyle deformation (*P* < 0.001) (Table 2). These variables were subsequently included in a multivariate analysis. Only the magnitude of setback (*P* = 0.032; odds ratio, 0.87; 95% confidence interval, 0.762–0.88) and the presence of TMJ symptoms (*P* = 0.007; odds ratio, 3.26; 95% confidence interval, 1.377–7.74) remained significant in the multivariate model (Table 3).

**Table 2:** Factors related to condylar sag (univariate analysis).

Variable	Category	TMJ dyslocation (−)	TNJ dyslocation (+)	*P*-value
Sex	Female	48	24	0.322
	Male	24	18	
Age		26.7 ± 10.8	28.0 ± 9.99	0.538
Side	Non-functional side	35	22	0.846
	Functional side	37	42	
TMJ symptoms	(−)	55	20	0.002
	(+)	17	22	
Wilkes classification	No findings	53	21	0.039
	Early	17	16	
	Early/intermediate	1	4	
	Intermediate/late	1	1	
Habitual luxation of TMJ	(−)	71	41	1.000
	(+)	1	1	
Maximal mouth opening	More than 30 mm	68	40	1.000
	30 mm or less	4	2	
Pain on mouth opening or closing	(−)	68	38	0.464
	(+)	4	4	
Mandibular condyle deformation	(−)	72	30	< 0.001
	(+)	0	12	
Maxillary surgery	(−)	42	20	0.331
	(+)	30	22	
Setback (mm)		6.61 ± 3.32	4.42 ± 3.64	0.001
Setback	Less than 3 mm	10	20	< 0.001
	3 mm or more and less than 6 mm	23	9	
	6 mm or more and less than 9 mm	25	10	
	9 mm or more and less than 12 mm	11	0	
	12 mm or more	3	3	
With occlusal cant correction	(−)	58	28	0.117
	(+)	14	14	
With occlusal cant correction (2 mm or more)	(−)	62	28	0.018
	(+)	10	14	
Method of bone incision	c-shaped	34	16	0.064
	Vertical	17	5	
	Oblique	21	21	

Values are expressed as mean ± standard deviation or the number of patients.

*TMJ* temporomandibular joint.

**Table 3:** Factors related to condylar sag (multivariate analysis).

Variable	*P*-value	OR	95% CI
Setback (mm)	0.032	0.868	0.762–0.88
TMJ symptoms, (−) versus (+)	0.007	3.264	1.377–7.740
Occlusal cant correction > 2 mm, (−) versus (+)	0.106	2.349	0.835–6.614

OR: odds ratio.

95% CI: 95% confidence interval.

TMJ: temporomandibular joint.

To quantify the relationship between the magnitude of setback and condylar sag after IVRO, a cut-off value was obtained from the receiver operating characteristic (ROC) curve using the Youden index as an indicator. Condylar sag after IVRO was significantly more likely in cases where the magnitude of setback was 3.25 mm or less.

## Discussion

The results of this study suggest that condylar sag after IVRO is associated with the magnitude of setback and TMJ symptoms. Therefore, it is important that these factors be evaluated during treatment planning for IVRO.

In this study, a decrease in the magnitude of setback was a significant predictor of condylar sag. It was considered that as the magnitude of setback decreased, the force with which the distal segment pushed the proximal segment backward decreased, and the condylar sag was more likely to occur. The association with TMJ symptoms is supported by the results of a prior study that reported a higher likelihood of condylar luxation in patients with TMD who experienced symptoms (such as clicking sounds and TMJ pain), compared to patients with simple jaw deformities^10^. These results may be attributed to an increased fragility and loss of elasticity in the joint capsule due to inflammation, which subsequently leads to condylar sag and luxation. The association between condylar sag and an occlusal cant correction of 2 mm or more may be explained by the development of TMD due to the perturbation of normal mandibular movement by the non-physiological inclination of the maxillary occlusal plane. This association was significant in the univariate analysis, but not in the multivariate analysis. Additional studies are required to confirm the association between TMJ symptoms and an occlusal cant correction of 2 mm or more.

While osteotomy line shape was not significantly associated with condylar sag, a significant relationship with luxation was reported in a previous study^11^. Additional studies are required to confirm this association between osteotomy line shape and condylar sag. Although mandibular condylar deformation was a significant explanatory variable in the univariate analysis, it was not retained in the multivariate model. This may be attributed to the finding that the exacerbation of symptoms in patients with TMD often results in mandibular condyle deformity. As a result, mandibular condyle deformation would not be evident prior to the appearance of TMJ symptoms. Therefore, new studies are needed to explore whether TMJ symptoms result in mandibular condylar deformity. In the ROC curve, the cut-off value for the magnitude of setback for condylar sag after IVRO was 3.25 mm; therefore, surgeons should be vigilant for condylar sag if the magnitude of setback is less than 3.25 mm.

A limited number of studies have reported on the development of condylar luxation (in which the condyle is postoperatively displaced from the glenoid fossa, beyond the articular eminence) from condylar sag. Yamauchi et al.^10^ reported that patients with TMD who have a setback magnitude of 0–1 mm frequently experienced condylar luxation. Campbell^15^ proposed two hypotheses for post-condylotomy changes, and stated that the condyle is more likely to be displaced anteriorly in patients after condylotomy compared to those with anterior disk displacement with reduction. Based on this, our perspective is that condylar luxation represents an exacerbation of condylar sag; therefore, by examining predisposing conditions for condylar sag, it may be possible to predict the risk of condylar luxation.

In this study, the medial pterygoid muscle was almost detached from the proximal segment. This facilitated the setback procedure and reduced the potential for possible relapse after IVRO. The complete detachment of the medial pterygoid muscle is typically avoided, as it may result in the complete displacement of the condyle from the glenoid fossa. Retaining some medial pterygoid muscle attachment also lowers the risk of condylar luxation and ischemic necrosis of the tip of the proximal segment^3,5,16^. Nevertheless, some studies have reported the absence of complications in cases where the entire medial pterygoid muscle was detached from the proximal segment^13,17^. In the present study, condylar sag was observed in just over a third (37%) of the cases, and only one case (0.87%) of condylar luxation was documented; the incidences of both complications are lower than that reported by previous studies^10,11^. Therefore, even if the medial pterygoid muscle is completely detached, the stability of the TMJ is relatively unaffected. In addition, avascular necrosis of the proximal segment was not observed in the postoperative 1-year follow-up period. This is notable, as the proximal segment was only connected to the mandibular condyle at the joint capsule. This implies that the blood supply and minimal amount of attachment provided by the joint capsule was sufficient to avoid avascular necrosis following IVRO. The adequacy of the blood supply from the joint capsule has been previously reported^17^.

Some limitations are acknowledged in the present study. First, it was a preliminary study involving a small number of patients. Second, we investigated the conditions under which condylar sag occurred, and subsequently inferred that these conditions would also apply to condylar luxation. Nevertheless, by taking into account the magnitude of setback during surgical planning, the presence of TMJ symptoms, and mandibular condyle deformation, it is possible to prevent condylar luxation, which is difficult to manage if it occurs. Further studies conducted across multiple sites are required to confirm the results of this study.

## Methods

### Subjects

The study protocol was approved by the Institutional Review Board of the Nagasaki University Graduate School of Biomedical Sciences (approval number 16020826), and all participants provided written informed consent, or informed consent was obtained from the parents or legally authorized representatives of subjects that are under 18. All experiments were performed in accordance with relevant guidelines and regulations. This retrospective cohort study included patients diagnosed with skeletal mandibular prognathism who underwent IVRO between November 2012 and February 2017 at the Unit of Translational Medicine in the Department of Clinical Oral Oncology at the Nagasaki University Graduate School of Biomedical Sciences. Inclusion criteria comprised of patients who (1) underwent mandibular setback via IVRO performed by the same surgeon, and (2) did not have previous orthodontic treatment. Patients were excluded if they did not agree to participate. We evaluated both the left and right TMJs in 57 patients (36 women and 21 men) using computed tomography (CT) (Aquilion 64™, Canon Medical Systems Corporation, Tochigi, Japan) before and after IVRO. This study utilized cases and methods that we have previously published^9^.

Three-dimensional (3D) CT images were used to diagnosis condylar sag and condylar luxation prior to surgery, and check for complications after surgery. The CT images were acquired with the mouth closed to ensure reproducibility of the distal segment position during diagnosis.

### Surgical procedure and MMF

IVRO was performed under general anesthesia. An intraoral incision was made at the anterior border of the ramus. A pair of Bauer retractors was placed in the sigmoid and antegonial notches to visualize the ante-lingular prominence, and to prevent bleeding from the internal maxillary artery. A subcondylar osteotomy was performed using an oscillating saw. The distal segment was slid distally and placed medially to the proximal segments. After IVRO, the medial pterygoid muscle was almost detached from the proximal segment in all cases. The distal fragment of the mandible was placed in the planned postsurgical position and stabilized using rigid MMF and a splint in the maxillary dental arch^9^. Rigid interosseous fixation was not performed in any of the patients. MMF was used for 3 days, after which guiding elastics were used for approximately 3 months.

### Assessment methods

**Figure 1:**
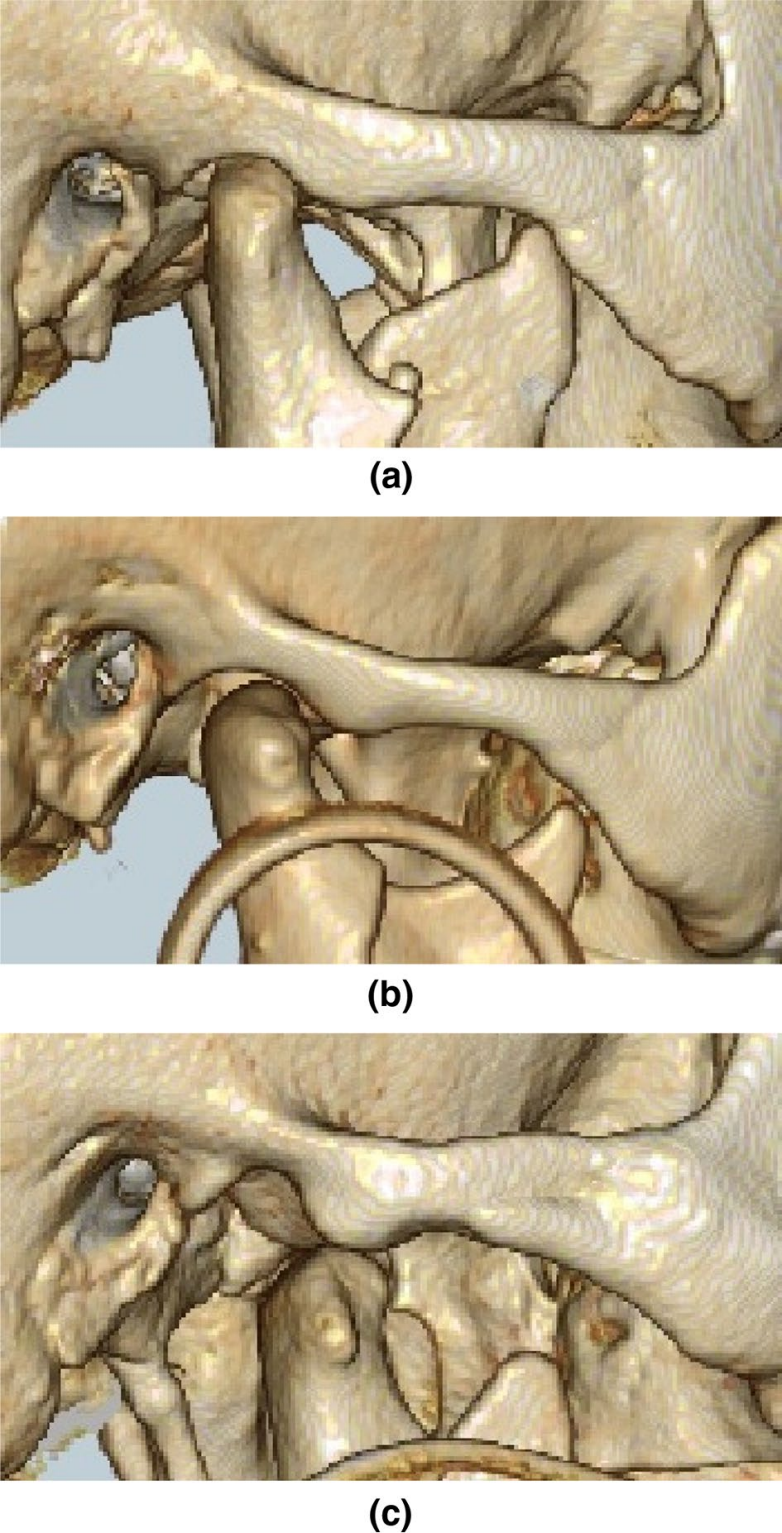
Diagnosis of temporomandibular joint sag and luxation. Illustrations showing (**a**) no sag of the temporomandibular joint, (**b**) sag of the temporomandibular joint, and (**c**) luxation of the temporomandibular joint.

Condylar sag and luxation after IVRO were diagnosed based on 3D-CT imaging on the first day after surgery of the left and right TMJs. A diagnosis of condylar sag was made if the highest point of the mandibular condyle was observed from the glenoid fossa. Condylar luxation was diagnosed when the mandibular condyle deviated from the glenoid fossa beyond the articular eminence (Fig. 1). All diagnoses were performed twice by three oral surgeons. Two or more oral surgeons were present during each diagnosis in order to ensure accuracy.

### Statistical analysis

In the univariate and multivariate analyses, condylar sag included all cases of condylar luxation, based on the view that condylar luxation is the result of condylar sag. Univariate and multivariate analyses were performed to determine the potential association of clinical factors with condylar sag and condylar luxation after IVRO. Condylar sag (or condylar luxation) after IVRO was the dependent variable, and the potential explanatory (independent) variables included the following: sex, age, magnitude of setback, classification of the main and sub-functional side of the TMJ (main functional side: ×, sub functional side: ◯), presence of TMJ symptoms (such as TMJ clicking, TMJ pain and trismus on mouth opening and closing, maximal mouth opening, habitual TMJ luxation), Wilkes classification, provision of concurrent maxillary surgery, occlusal cant modification, magnitude of occlusal cant modification (≥ 2 mm or < 2 mm), presence of mandibular condyle deformation, and an osteotomy line shape. For factors found to be significantly associated with condylar sag or condylar luxation during multivariate analysis, cut-off values were determined from the ROC curve. All statistical analyses were performed with SPSS software (version 24.0; Japan IBM Co., Tokyo, Japan). A *P*-value less than 0.05 was considered statistically significant.

## Data Availability

All data generated or analyzed during this study are included in this published article.
